# Integrating Epistatic Interactions into Genomic Prediction of Growth and Fillet Fat Content in Common Carp

**DOI:** 10.3390/ijms27188315

**Published:** 2026-09-18

**Authors:** Ruixin Zhang, Xiaoyue Zhu, Zhipeng Sun, Xianhu Zheng, Yongjun Shu, Guo Hu

**Affiliations:** 1 Key Laboratory of Freshwater Aquatic Biotechnology and Breeding, Ministry of Agriculture and Rural Affairs, Heilongjiang River Fisheries Research Institute, Chinese Academy of Fishery Sciences, Harbin 150070, China; zrxin2001@126.com (R.Z.); zhuxiaoyue2001@126.com (X.Z.); sunzhipeng@hrfri.ac.cn (Z.S.); zhengxianhu@hrfri.ac.cn (X.Z.); 2 Key Laboratory of Molecular Cytogenetics and Genetic Breeding of Heilongjiang Province, College of Life Science and Technology, Harbin Normal University, Harbin 150025, China

**Keywords:** epistasis, genomic prediction, AlphaFold2, SNP reduction, driver interaction pairs

## Abstract

Common carp (*Cyprinus carpio*) is an important freshwater aquaculture species, yet non-additive genetic effects remain poorly understood for economically important traits. Here, we dissected epistatic interactions affecting standard body length (SL) and fillet fat content (FC) by integrating pathway-level epistasis analysis, genomic selection (GS), and AlphaFold2 (AF2)-based structure prediction. BridGE analysis detected extensive epistatic signals for both traits. Incorporating selected interactions into GS models improved predictive ability within the sampled population, yielding correlations of 0.85 for SL and 0.87 for FC. An epistasis-informed reduction strategy downsized the initial 2.2 million SNPs to 12,902 candidate markers while retaining useful predictive information. AF2-based predictions provided complementary structural support for a subset of candidate driver interactions. Network and enrichment analyses further revealed distinct molecular features associated with SL and FC. Together, these findings suggest that incorporating epistatic information can improve genomic predictive ability of complex traits in common carp, and provide a basis for further evaluation of epistasis-informed selection in independent aquaculture populations.

## 1. Introduction

Common carp (*Cyprinus carpio*) is an important and widely cultured freshwater aquaculture species in China and is also one of the core aquaculture species in Central and Eastern Europe [1,2]. Owing to its broad ecological adaptability and high-quality flesh, common carp plays an important role in ensuring the supply of high-quality animal protein. With the continuous growth of the global population, which is projected to approach 10 billion by 2050, the demand for high-quality protein is expected to increase substantially [3]. Consequently, promoting the development of aquaculture toward higher efficiency and improved product quality has become an inevitable trend. In this context, elucidating the genetic basis of economically important traits in common carp is of great significance for accelerating genetic improvement. Standard body length (SL), as a key indicator of growth performance, is directly associated with aquaculture profitability, whereas fillet fat content (FC) is one of several factors contributing to flesh quality and may also influence the commercial value of fish products [4,5]. Therefore, genetic improvement studies focusing on body length and fat content can not only enhance the production efficiency of common carp aquaculture but also provide scientific support for the stable supply of high-quality protein.

Over the past two decades, rapid advances in molecular breeding technologies have led to a series of important achievements in common carp genetics and genomic selection, laying a solid foundation for precision breeding [6]. Genomic selection (GS), first proposed by Meuwissen et al. in 2001, has become an important tool in modern breeding and is now widely applied in both plant and animal breeding programs [7]. Compared with traditional phenotype-based selection methods, GS uses genome-wide single-nucleotide polymorphism (SNP) markers to predict genomic estimated breeding values (GEBVs), thereby substantially shortening breeding cycles and improving selection accuracy [8]. In recent years, with the reduction in sequencing costs, GS has shown strong potential in the improvement of various aquaculture species and traits, including Atlantic salmon and large yellow croaker [9,10]. In common carp, GS has also been demonstrated to be effective for improving multiple traits. For example, Palaiokostas et al. (2018) [11] reported that the use of the GBLUP method increased the accuracy of breeding value prediction for growth traits in juvenile carp by 18% compared with traditional pedigree-based approaches. Similarly, Sun et al. (2025) [12] demonstrated that GS can be effectively applied to the genomic prediction and genetic improvement of fillet fat content in common carp, with predictive ability reaching as high as 0.95 for this trait. However, large-scale implementation of GS in aquaculture still faces two major challenges. On the one hand, there is a contradiction between the high cost of high-density SNP genotyping and the relatively low economic value of individual aquatic animals [13]. On the other hand, most current studies focus on additive genetic effects, while non-additive components remain insufficiently explored, limiting further improvements in predictive ability.

Epistasis, as a core component of non-additive genetic effects, refers to the nonlinear genetic interactions between non-allelic genes that influence the same trait [14,15]. Since the concept was first proposed, numerous studies in model organisms and crop breeding have demonstrated that epistatic interactions are widely present in key traits related to growth, quality, and stress resistance in both animals and plants [16,17,18]. Previous studies have shown that incorporating epistatic effects into genomic selection models can effectively compensate for the limitations of traditional additive models and improve the predictive ability of complex traits. For example, He et al. (2016) [19] reported that introducing epistatic effects in winter wheat breeding increased predictive ability by approximately 5%. Similarly, Chen et al. (2025) [20] demonstrated in Large White pigs that incorporating epistatic effects improved the predictive ability of total number born by 1.78%. Physical interactions between proteins constitute an important molecular basis of genetic epistasis. AlphaFold2 (AF2), as a high-precision protein structure prediction tool, enables efficient inference of protein interaction interfaces and binding affinity characteristics, thereby providing technical support for genome-wide identification of epistatic loci mediated by protein interactions [21,22,23]. Previous studies have demonstrated that the strength of epistatic effects is significantly associated with molecular features of protein interactions, such as binding free energy and stoichiometric ratios [24]. The application of AF2 has made it possible to systematically explore and quantitatively evaluate these features. However, in current studies on the genetic improvement of common carp, the exploration of epistatic effects remains relatively limited. Not only are systematic analyses of epistasis scarce, but studies integrating epistatic effects into GS models have not yet been reported. Moreover, no studies have yet attempted to investigate epistatic effects in common carp using AF2. Therefore, a deeper understanding of non-additive genetic effects and their integration into predictive models represents a key step toward improving the predictive ability of genomic selection and enhancing breeding efficiency in common carp.

In this study, standard body length and fillet fat content in common carp were selected as target traits to systematically investigate the role of epistatic effects in the genetic regulation of complex traits and to explore optimized GS strategies that balance predictive ability and genotyping cost. Through genome-wide epistasis detection, key interacting loci associated with these traits were identified. On the one hand, the corresponding non-additive genetic effects were incorporated into six GS models to quantitatively evaluate the extent to which epistatic interactions improve predictive ability. On the other hand, epistasis signals were utilized to achieve efficient SNP marker reduction, allowing the performance of GS under reduced marker density to be assessed. Furthermore, this study innovatively applied AF2 to perform three-dimensional structural prediction of the identified driver interaction pairs. By analyzing the predicted structural features of candidate protein pairs, we sought to provide computational structural support for a subset of the identified interactions and assess their potential for protein–protein interactions. The findings will clarify the contribution of non-additive genetic effects to growth and quality traits in common carp. They will also provide a GS framework for aquaculture species that simultaneously considers predictive ability and cost efficiency.

## 2. Results

### 2.1. Phenotypic Distribution and Correlation Analysis

**Figure 1:**
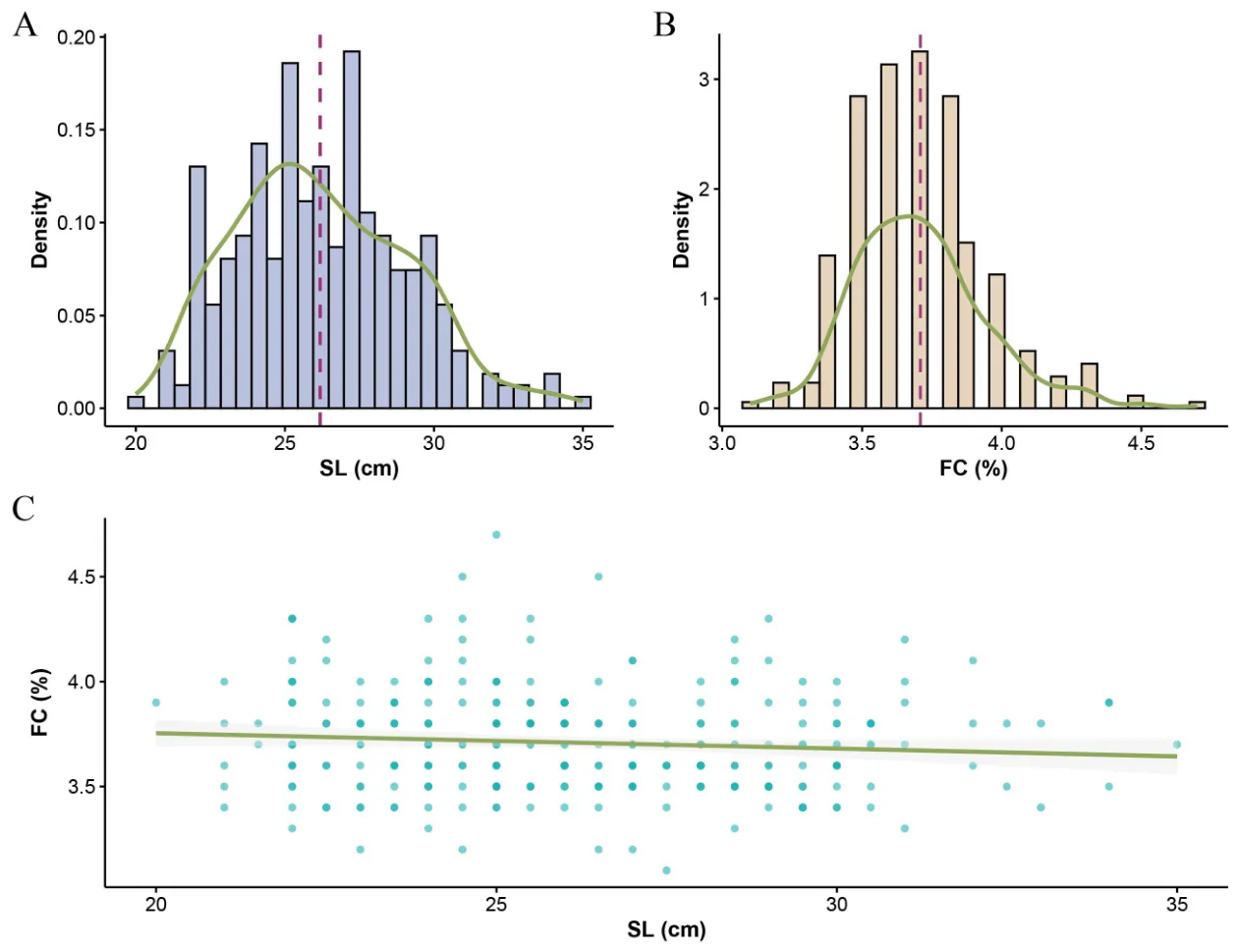
Phenotypic characterization of standard body length and fillet fat content in common carp. (**A**,**B**) Frequency distribution histograms of standard body length (SL) and fillet fat content (FC). The purple dashed line indicates the mean, and the green curve represents the density estimation (*n* = 312). (**C**) Scatter plot showing the correlation between SL and FC. The green line represents the linear regression fit, and the gray shaded area indicates the 95% confidence interval.

To investigate the genetic variation characteristics of body length and fillet fat content in the carp population, statistical analyses were conducted on the phenotypic distributions of SL and FC, as well as their interrelationship. The results showed that the mean value of SL was 26.19 cm with a standard deviation of 2.88 cm. Its distribution curve was symmetric around the mean (Figure 1A) and approximated a normal distribution, indicating that the samples exhibited relatively balanced variation in longitudinal growth and development. The mean value of FC was 3.71% with a standard deviation of 0.24, and its distribution also approximated a normal pattern (Figure 1B), suggesting that the variation of FC among individuals was relatively stable. Pearson correlation analysis was further conducted to evaluate the linear relationship between the two traits (Figure 1C). No significant linear correlation was detected between SL and FC (r = −0.0896, *p* = 0.114), indicating that no evident linear association exists between SL and FC in this carp population. Therefore, whether these two traits are regulated by distinct genetic mechanisms remains to be further investigated.

### 2.2. Heritability Analysis

Before conducting genomic selection analyses, GCTA was used to estimate the heritability of SL and FC in 312 carp individuals, aiming to quantify the contribution of additive genetic effects to the target traits (Table 1). The results showed that SL exhibited a moderate and significant additive genetic contribution, with a genome-wide narrow-sense heritability ($h^{2}$) of 0.34 (*p* = 0.01). The corresponding additive genetic variance ($\sigma_{g}^{2}$) and residual environmental variance ($\sigma_{e}^{2}$) were 2.79 and 5.49, respectively. In contrast, for FC, the estimated additive genetic variance reached the boundary of the parameter space ($\sigma_{g}^{2}$ = 3.48 × 10^−18^ ± 0.01), causing the heritability estimate to converge to 0.00, with no statistical significance (*p* = 1.00). This boundary solution indicates limited power to detect small additive contributions rather than a true absence of additive genetic variation.

### 2.3. Epistasis Analysis of Genomic Selection Models

**Figure 2:**
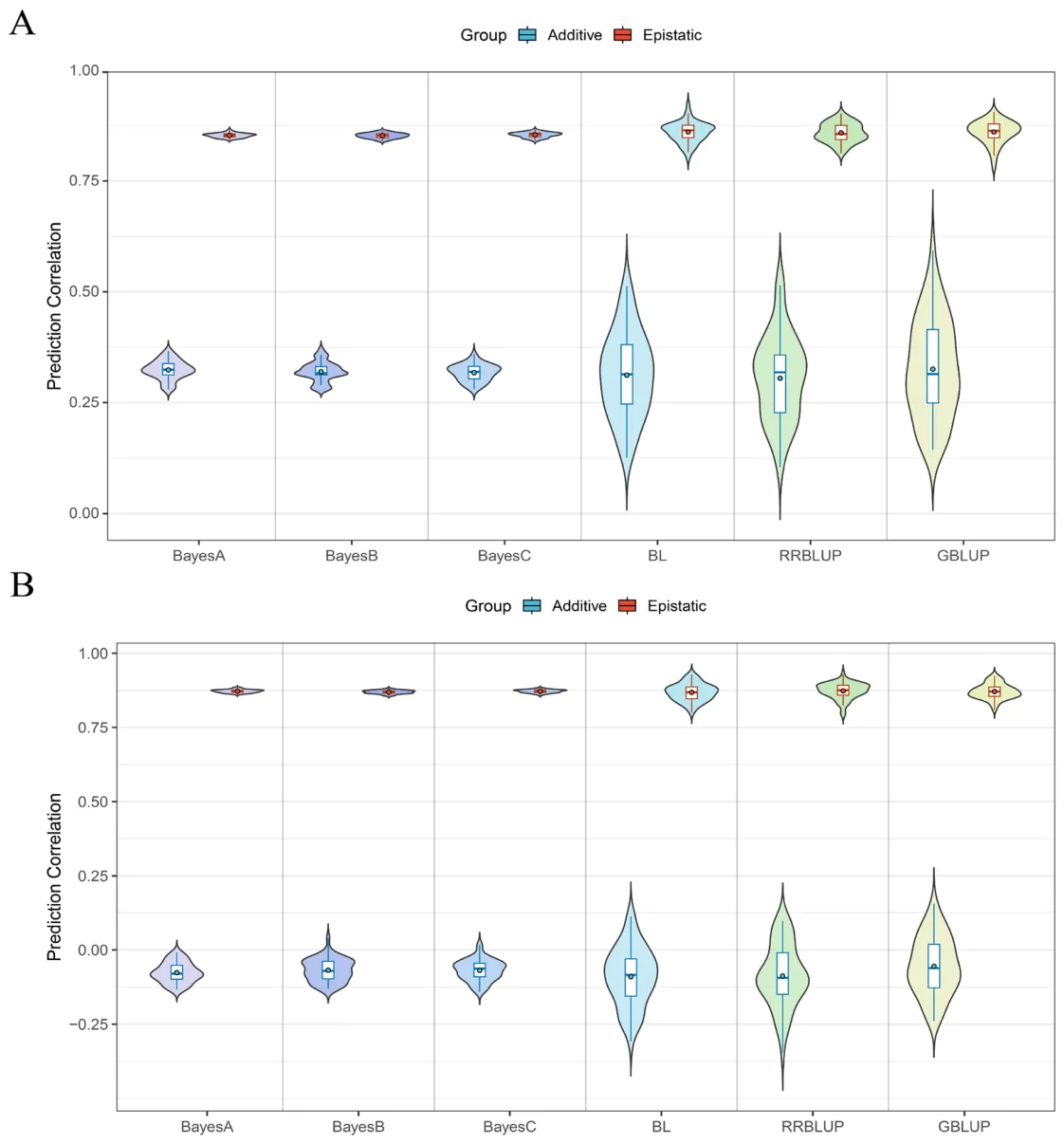
Comparison of predictive ability across different genomic selection (GS) models for standard body length and fillet fat content. (**A**,**B**) Predictive ability of six GS models (BayesA, BayesB, BayesC, BL, RRBLUP, GBLUP) under two effect frameworks for standard body length (SL) and fillet fat content (FC). “Additive” represents baseline models including only additive effects, while “Epistatic” represents models incorporating epistatic SNP interactions. Blue and red boxplots indicate the Additive and Epistatic groups, respectively. The outer shapes of the violin plots show the full distribution of predictive correlations across 50 replicates (*n* = 312); the inner boxplots denote the median and interquartile range.

Through epistatic signal screening using the BridGE algorithm, the initial 2,207,321 genome-wide SNP markers were reduced to 12,902 markers, representing a reduction of more than 99%. Based on the favourable candidate SNP interaction pairs identified by BridGE, 1350 and 848 SNP pairs were integrated for SL and FC, respectively. We compared the predictive performance of six genomic selection models under an additive-only framework (Additive group) and an additive plus epistasis framework (Epistatic group). The results showed that incorporating epistatic interactions improved the predictive ability for both traits (Figure 2). For SL, predictive ability under additive models ranged from 0.31 to 0.33, whereas after incorporating epistasis, predictive ability increased to 0.85–0.86 across all six models (Figure 2A). Among them, the Bayes LASSO model achieved the best performance, with predictive ability reaching 0.8586 in the epistatic model, compared to 0.3174 in the additive model, representing an increase of 0.5412. For FC, additive models generally yielded negative predictive ability (−0.0765 to 0.0569). After integrating epistatic interactions, predictive ability increased to approximately 0.87 across all models, with an absolute increase ranging from 0.9297 to 0.9489 (Figure 2B). The largest gain was observed in the BayesA model, where predictive ability increased from −0.0765 to 0.8724. Additional performance metrics, including RMSE, regression slope, and pairwise model comparisons by Tukey’s HSD, are shown in Figure S1. Under the additive model, ten random subsets of 12,902 SNPs yielded mean predictive abilities of 0.3121 ± 0.0052 for SL and −0.1055 ± 0.0118 for FC. The single epistasis-informed panel produced predictive abilities of 0.3261 for SL and −0.0569 for FC.

### 2.4. AlphaFold2 Prediction of Driver Interaction Pairs

**Figure 3:**
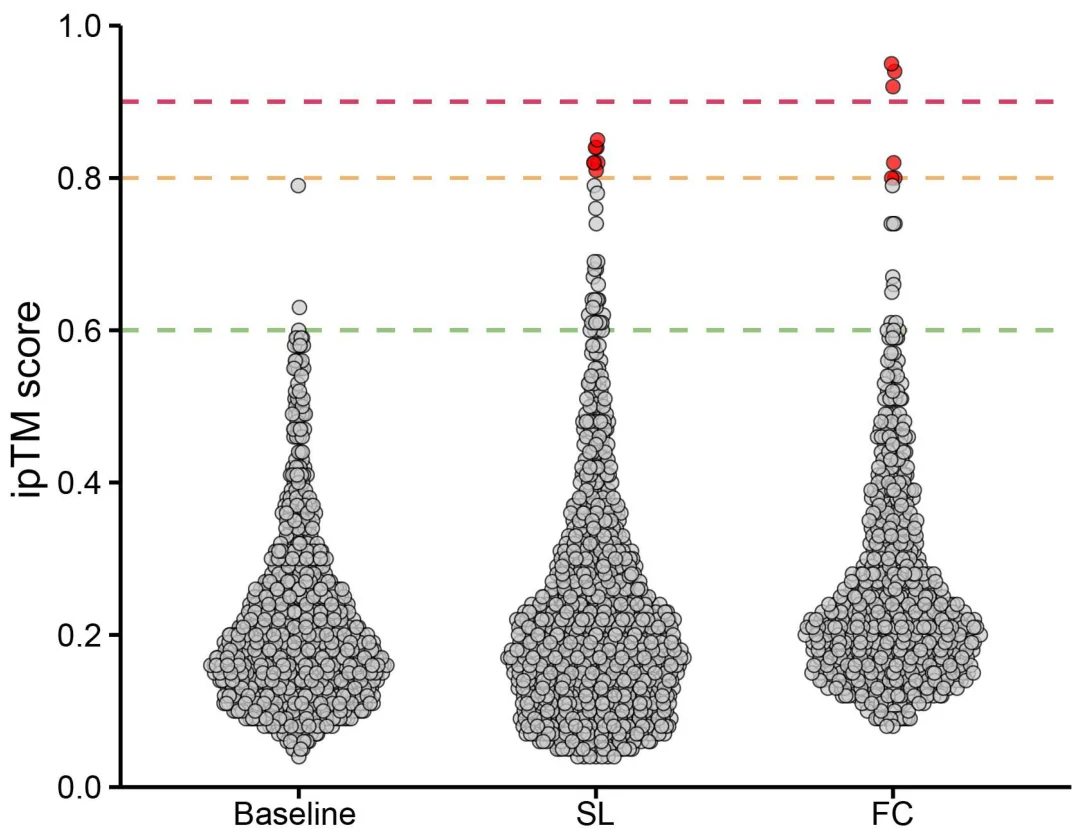
Distribution of ipTM scores for driver interaction protein pairs predicted by AlphaFold2. Violin plots show the distribution of interface predicted template modeling (ipTM) scores for the size-matched baseline group, standard body length (SL)-related group, and fillet fat content (FC)-related group. Green, orange, and red dashed lines indicate ipTM thresholds of 0.6 (permissive screening cutoff), 0.8 (high confidence), and 0.9 (very high confidence), respectively. Red dots highlight high-quality protein interaction predictions with ipTM ≥ 0.8. SL, *n* = 983 successfully modeled pairs; FC, *n* = 640 successfully modeled pairs; Baseline control, *n* = 992 successfully modeled pairs.

Epistasis analysis identified 1297 and 652 driver interaction pairs for the SL and FC traits, respectively. Among these, AF2 was used to predict protein–protein interactions for SL-related pairs (1000 pairs), FC-related pairs (652 pairs), and size-matched baseline pairs (1000 pairs). The number of successfully modeled interaction pairs was 983 for SL, 640 for FC, and 992 for the control group. An ipTM ≥ 0.6 threshold served as our permissive screening cutoff. Using this threshold, we identified 35 interacting protein pairs in the SL group and 18 in the FC group, compared with only 3 pairs in the control group (Figure 3, Table S2). When the threshold was increased to ipTM ≥ 0.7, 11 pairs remained in SL, 10 in FC, and only 1 in the control group. These predictions were considered candidate or intermediate-confidence structural associations. At the more stringent threshold of ipTM ≥ 0.8, 7 SL pairs and 6 FC pairs showed stronger computational structural support, whereas no such interactions were detected in the control group. Fisher’s exact tests further indicated that the number of interacting pairs was significantly higher in the SL and FC groups than in the size-matched baseline group across all thresholds (Table S3). Collectively, the interaction pairs identified by BridGE showed a higher frequency of predicted protein–protein associations than the size-matched baseline pairs, and a subset attained higher ipTM scores. These findings provide complementary structural evidence for a subset of candidate interaction pairs. They suggest that some corresponding genes may form potential protein-level associations relevant to the genetic regulation of SL and FC.

### 2.5. Three-Dimensional Structural Characterization of Protein Interaction Pairs

**Figure 4:**
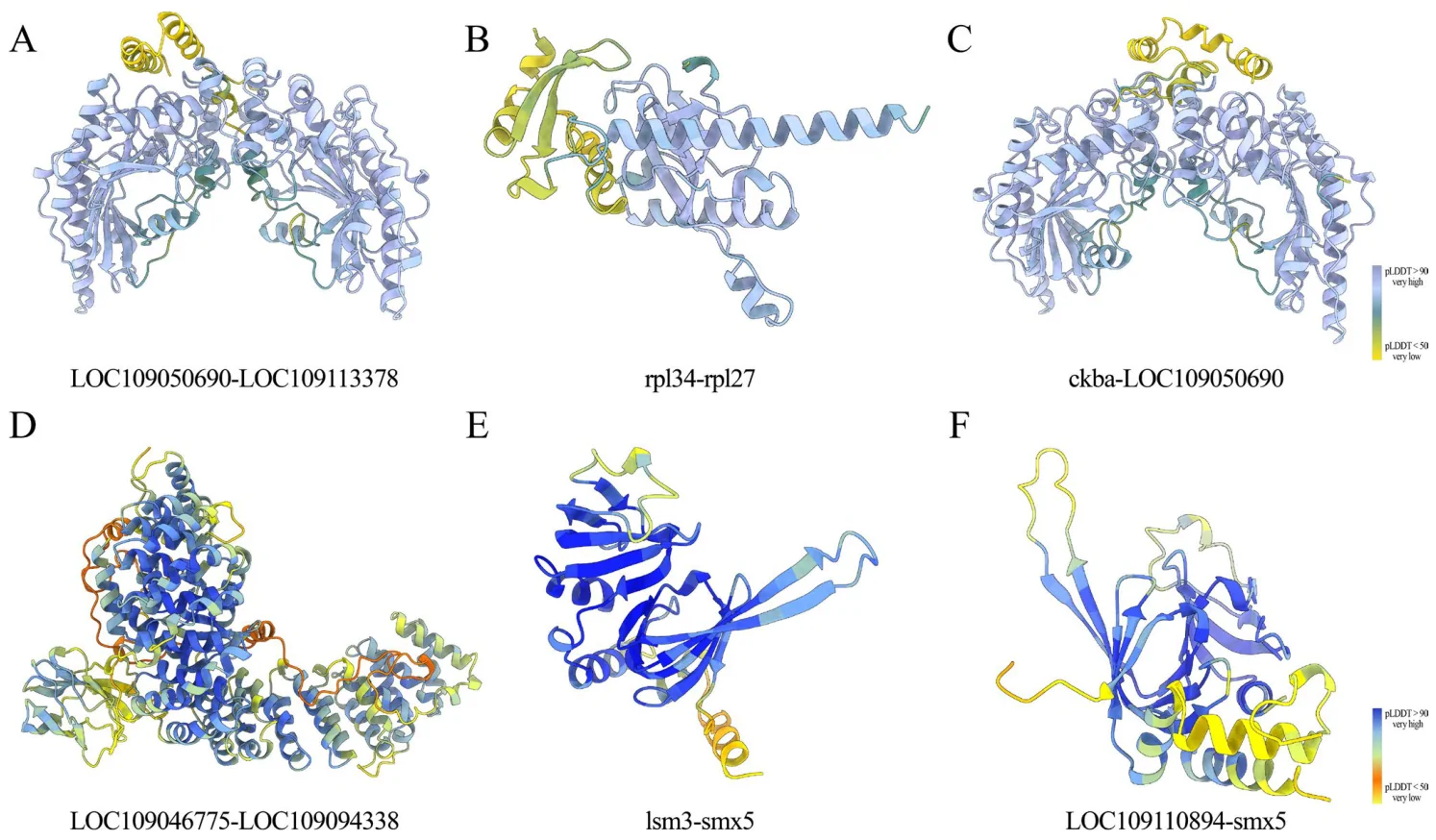
Three-dimensional structures of six high-confidence heterodimers predicted by AlphaFold2. (**A**–**C**) Structural visualization of standard body length (SL)-related dimers: LOC109050690-LOC109113378, rpl34-rpl27, and ckba-LOC109050690. (**D**–**F**) Structural visualization of fillet fat content (FC)-related dimers: LOC109046775-LOC109094338, lsm3-smx5, and LOC109110894-smx5. Protein backbones are colored based on predicted local distance difference test (pLDDT) scores. The color bar illustrates pLDDT scores in the range of 0–100. Blue (pLDDT > 90) represents very high confidence; a pLDDT score of 70–90 represents good confidence; 50–70 represents low confidence; and yellow (pLDDT < 50) represents very low confidence, corresponding to potential disordered regions.

To further elucidate the structural characteristics of protein interactions associated with SL and FC traits, six high-confidence interaction pairs predicted by AF2 were visualized and analyzed in three dimensions using ChimeraX. The results showed that all six heterodimeric complexes demonstrated high structural confidence and strong interface binding properties (Figure 4). Among the SL-related protein interactions, the pairs LOC109050690-LOC109113378, rpl34-rpl27, and ckba-LOC109050690 showed predicted local distance difference test (pLDDT) scores of 85.58, 77.39, and 85.65, respectively, with corresponding buried surface areas (BSA) of 1631 Å^2^, 1197 Å^2^, and 2023 Å^2^ (Figure 4A–C). For the FC-related protein interactions, the pairs LOC109046775-LOC109094338, lsm3-smx5, and LOC109110894-smx5 exhibited pLDDT scores of 78.99, 88.00, and 84.95, with BSA values of 4771 Å^2^, 1202 Å^2^, and 1152 Å^2^, respectively (Figure 4D–F). In terms of spatial structural features, all six complexes formed interaction interfaces primarily composed of typical α-helices and β-sheets. Notably, the BSA values of all interaction pairs exceeded 1000 Å^2^, meeting the threshold commonly associated with biologically specific interactions. Combined with the pLDDT scores and BSA measurements, these results indicate that the identified interaction pairs possess reliable structural conformations. They also provide structural evidence for their potential functional roles in regulating growth and lipid metabolism in common carp.

### 2.6. Protein–Protein Interaction Network Analysis

**Figure 5:**
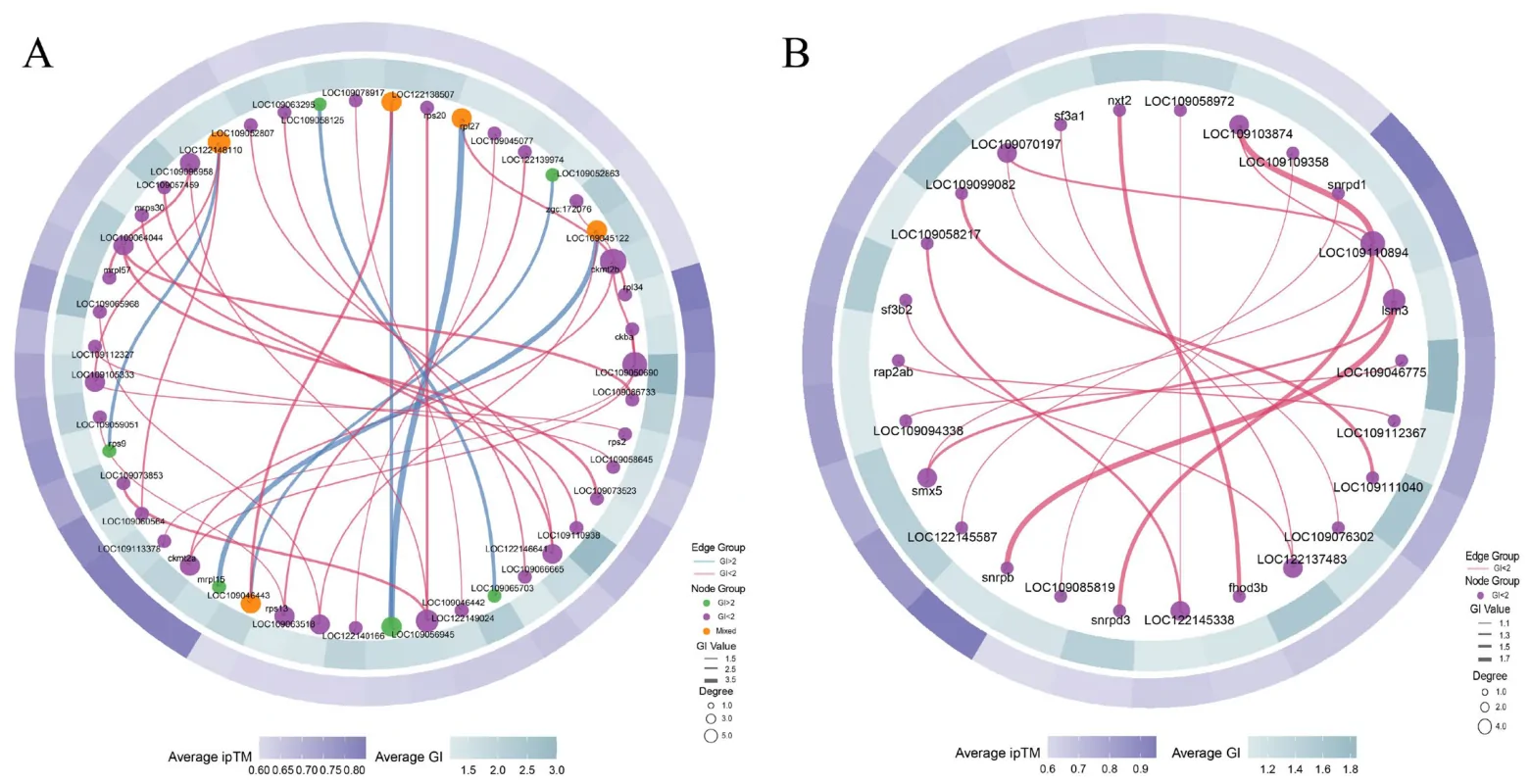
Protein interaction network analysis. (**A**,**B**) Protein interaction networks for standard body length (SL) and fillet fat content (FC) traits. The inner topology represents interactions filtered with interface predicted template modeling (ipTM) ≥ 0.60. Nodes represent interacting genes, with node size proportional to connectivity degree. Node colors indicate their distribution in genetic interactions (GI) (green: GI > 2; purple: GI < 2; orange: shared genes). Edges represent physical interaction strength, with line width proportional to GI values; blue and pink edges correspond to different GI thresholds. The outer circular heatmap integrates average effect values, where cyan gradients indicate average GI values and purple gradients indicate ipTM confidence levels.

Based on AF2-predicted protein interaction pairs with potential physical interaction capability, PPI networks associated with SL and FC traits were constructed. As shown in Figure 5, the SL-related network consisted of 47 nodes and 35 edges (Figure 5A). Among these, the interaction pair LOC109050690-LOC109113378 exhibited the highest physical interaction confidence (ipTM = 0.85), while rpl27-LOC109050690 showed the strongest genetic interaction effect (GI = 3.532). Topological analysis of the network revealed that the gene *LOC109050690* (encoding creatine kinase) had the highest connectivity and overall effect. This gene participates in energy metabolism by catalyzing the conversion between ATP and phosphocreatine. In the FC-related network (Figure 5B), a total of 26 nodes and 18 edges were identified. The interaction pair LOC109046775-LOC109094338 showed the highest ipTM value (0.95), while lsm3-snrpb exhibited the most significant genetic interaction effect (GI = 1.843). The gene *LOC109110894* was identified as the hub gene with the highest connectivity in this network and encodes the Sm D2 protein, a component of the spliceosome. Further integrated analysis using the outer circular heatmap showed the distribution of genetic interaction signals and predicted structural information across the two networks. Both networks contained interaction modules with simultaneously high GI and ipTM values. The integration of genetic interaction signals and predicted structural information provides complementary evidence for prioritizing candidate interaction pairs identified by the BridGE analysis.

### 2.7. Functional Enrichment Analysis of Driver Interaction Genes

**Figure 6:**
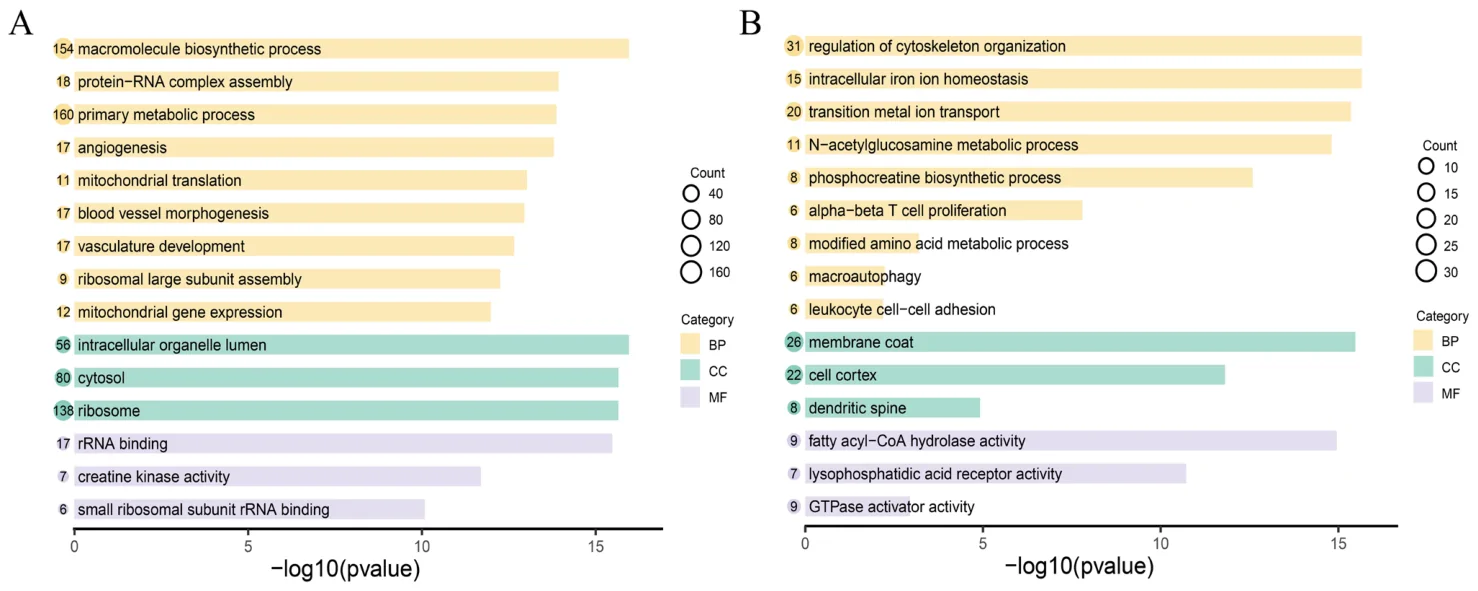
GO functional enrichment analysis of interaction genes associated with standard body length and fillet fat content in common carp. (**A**,**B**) GO enrichment results for standard body length (SL)- and fillet fat content (FC)-related genes. The x-axis represents −log10(*p*-value), indicating enrichment significance. Numbers on the left indicate the number of genes enriched in each term, and bubble size is proportional to gene count. Categories include biological process (BP, yellow), cellular component (CC, green), and molecular function (MF, purple). Results are simplified by merging terms and removing redundancy (Fisher’s exact test, FDR-adjusted *p* < 0.05).

To investigate the biological functions associated with interaction genes related to SL and FC in common carp, Gene Ontology (GO) enrichment analysis was performed on genes involved in the driver interaction pairs. The results revealed a clear functional divergence between SL and FC traits at the gene function level. Genes associated with the SL trait were significantly enriched in functional modules closely related to cell growth and metabolism (Figure 6A). In the biological process (BP) category, these genes were mainly involved in macromolecule biosynthesis, protein–RNA complex assembly, mitochondrial translation, and vascular development processes (such as angiogenesis and vasculature morphogenesis). In terms of cellular components (CC), these functions were primarily localized to intracellular organelle lumens, the cytoplasm, and ribosomes. At the molecular function (MF) level, significant enrichment was observed for rRNA binding, small ribosomal subunit rRNA binding, and creatine kinase activity (corresponding to the biochemical function of the hub gene *LOC109050690* identified above). In contrast, genes associated with the FC trait were mainly enriched in functional modules related to lipid-associated processes, cytoskeletal dynamics, and ion homeostasis (Figure 6B). At the BP level, these genes were significantly involved in regulation of cytoskeleton organization, intracellular iron ion homeostasis, N-acetylglucosamine metabolism, and phosphocreatine biosynthesis. At the CC level, they were primarily localized to membrane coats and the cell cortex. At the MF level, significant enrichment was observed for fatty acyl-CoA hydrolase activity and lysophosphatidic acid receptor activity. These enrichment results offered suggestive evidence for the driver interaction genes associated with SL and FC.

### 2.8. Association Analysis Between Two-Locus Genotype Combinations and Phenotypes

**Figure 7:**
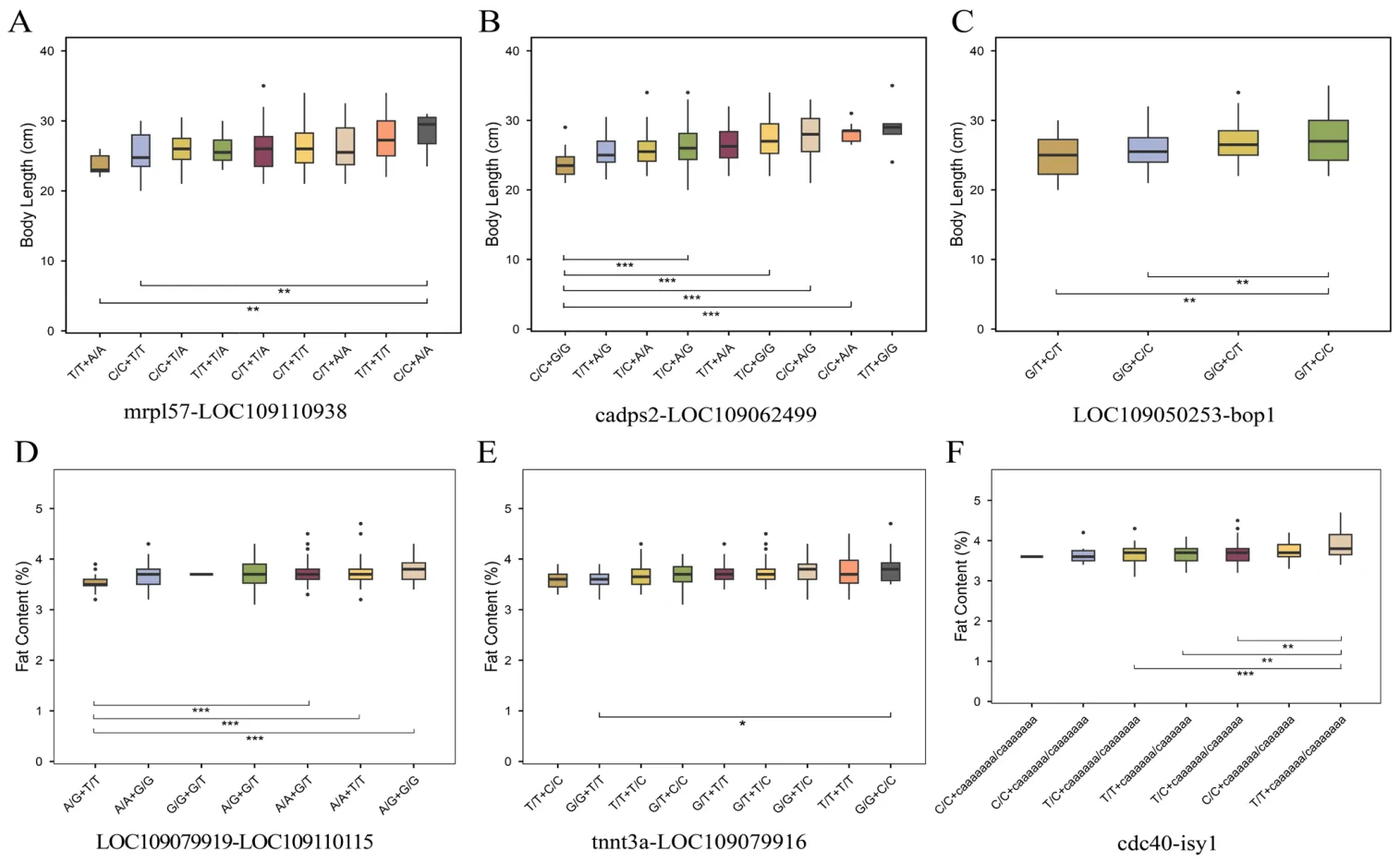
Association analysis between two-locus genotype combinations of key interaction pairs and standard body length and fillet fat content traits. (**A**–**C**) Two-locus genotype combination analysis of standard body length (SL)-related interaction pairs, with the y-axis representing body length (cm). (**D**–**F**) Two-locus genotype combination analysis of fillet fat content (FC)-related interaction pairs, with the y-axis representing fat content (%). Boxplots show phenotypic distributions under different genotype combinations, with genotype combinations labeled on the x-axis. Asterisks indicate statistical significance between groups (* *p* < 0.05, ** *p* < 0.01, *** *p* < 0.001).

Phenotypic variation across two-locus genotype combinations was assessed using three representative interaction pairs per trait (Figure 7). For SL-related pairs, joint genotypes strongly influenced body length. In mrpl57–LOC109110938, T/T + A/A was linked to shorter length (mean 23.79 cm), while C/C + A/A reached 28.53 cm. Similarly, cadps2–LOC109062499 displayed contrasting outcomes: C/C + G/G gave a shorter length (23.67 cm), whereas T/T + G/G increased it to 29.10 cm. For LOC109050253–bop1, the G/T + C/T and G/T + C/C combinations resolved into distinct short (25.03 cm) and long (27.03 cm) phenotypes. FC-related pairs exhibited comparable trends. In LOC109079919–LOC109110115, fat levels dropped to 3.52% under A/G + T/T but rose to 3.80% under A/G + G/G. The tnnt3a–LOC109079916 pair also separated low-fat (T/T + C/C) and high-fat (G/G + C/C) groups. Lastly, the cdc40–isy1 pair recorded its lowest fat content (3.60%) under C/C + caaaaaaaa/caaaaaaa, compared to 3.91% under T/T + caaaaaaaa/caaaaaaa. In summary, candidate two-locus genotype combinations effectively separated phenotypic extremes in this population. These findings support the incorporation of epistatic interaction terms into genomic prediction frameworks.

## 3. Discussion

In this study, we explored whether epistatic information could improve genomic prediction for complex traits in common carp. Although conventional additive models showed limited predictive ability for some traits, incorporation of the selected epistatic interaction terms increased predictive ability for both traits under the present analytical framework. For SL trait, predictive correlation increased from 0.32 to 0.85, representing an absolute gain of roughly 0.54. The contrast was even more pronounced for FC trait, where additive models were nearly ineffective; including epistatic effects raised predictive ability from −0.07 to 0.87. These gains suggest that non-additive interaction terms capture substantial predictive information omitted by additive markers alone. The distinct gains between traits likely reflect their underlying genetic architectures. SL, as a structural trait reflecting skeletal development, is more stably regulated by additive genetic effects [25]. In contrast, FC, as a metabolic trait, is highly susceptible to environmental factors such as feeding conditions, stress, and sampling location, leading to dilution of additive genetic signals by environmental noise [26]. The enrichment of creatine kinase activity and mitochondria-related functions among SL-associated interaction genes may point to potential links with energy metabolism (Figure 6A). Similarly, the enrichment of spliceosome-related functions among FC-associated genes may suggest a connection with RNA processing (Figure 6B). Incorporating non-additive terms may thus help recover predictive information for traits with weak additive signals, though the proportion of phenotypic variance directly attributable to epistasis remains to be quantified.

The large-scale application of genomic selection in aquaculture has long been constrained by the trade-off between SNP genotyping costs and predictive ability. While high-density SNP panels improve the capture of genetic variation, they significantly increase sequencing and computational costs. Conversely, improperly selected low-density panels may lead to reduced predictive ability [27,28]. To construct cost-effective SNP subsets, previous studies have often relied on GWAS to identify trait-associated loci, such as 12K SNP panels in black carp and 5K SNP panels in Malabar red snapper [29,30]. However, such approaches are highly dependent on the strength of trait-association signals, are sensitive to population structure and genetic architecture, and are limited in capturing non-additive genetic effects. In this study, we adopted the default pipeline of the BridGE epistasis detection algorithm to implement an SNP reduction strategy without prior GWAS screening. Based solely on epistatic interaction signals, the original 2,207,321 SNPs were efficiently reduced to 12,902, decreasing marker density from the million scale to the ten-thousand scale. This outcome provides a candidate analytical framework for reduced-density genotyping.

This marker-reduction strategy provides two practical considerations. First, the resulting marker density of approximately 13K is within the range of reduced-density panels evaluated in other aquaculture species [29,31]. Second, marker selection and identification of candidate non-additive features were performed within the same analytical framework. In an equal-density comparison under GBLUP, the epistasis-informed panel showed additive predictive ability broadly comparable to the random 12,902-SNP panels (SL: 0.3261 vs. 0.3121 ± 0.0052; FC: −0.0569 vs. −0.1055 ± 0.0118). Thus, the marker reduction did not substantially compromise the baseline additive predictive performance observed in this cohort. At the same time, the comparison provides only limited evidence that the epistasis-informed strategy outperforms a size-matched random panel. The major predictive differences between the additive and epistatic models therefore arose from the explicit inclusion of the selected interaction terms rather than from marker reduction alone. This framework may serve as a basis for further evaluation of reduced-density genotyping combined with epistatic information in aquaculture populations.

Epistasis is often regarded in genetics as a statistical representation of nonlinear interactions between genes. Whether such interactions have a structural basis is an important criterion for evaluating the reliability of genetic models [32]. In this study, we employed AF2 protein structure prediction to provide structural support for driver interaction pairs from a structural biology perspective [33]. Across different ipTM thresholds, the frequency of PPI detected in the SL and FC experimental groups was consistently and significantly higher than that in baseline control groups (Table S3, Figure 3). This consistent enrichment trend provides additional computational evidence for the genetic signals identified by the BridGE algorithm. Notably, in the FC trait with low additive variance, AF2 still identified multiple high-confidence (ipTM ≥ 0.8) protein–protein interactions. This finding is consistent with previous observations in systems characterized by weak additive signals (e.g., SARS-CoV-2 variants) [34]. While single-locus additive effects may be masked by environmental noise, stable molecular interactions formed through protein binding may constitute a potential mechanistic basis underlying complex phenotypic variation [24,35]. By establishing a logical chain linking statistical epistasis to the prediction of protein–protein interactions, this study demonstrates the potential value of incorporating structural biology information into breeding value assessment systems [36]. However, AF2 confidence metrics alone cannot establish in vivo protein interactions or their relevance to trait regulation [37]. The structural predictions should therefore be regarded as a prioritization approach. Combining structural prediction with statistical epistasis may help prioritize candidate interactions for further investigation rather than establish a direct molecular mechanism.

Several methodological considerations qualify these findings. Pre-selecting epistatic features across the full dataset prior to cross-validation may introduce optimistic bias relative to fold-nested selection [38]. Furthermore, fitting interaction terms alongside low-density markers in 312 individuals presents a high predictor-to-sample ratio, compounding overfitting risks. These results should therefore be interpreted as internal estimates of predictive capacity within the current cohort. Validation in independent populations using nested feature selection will be important before practical implementation in breeding programs.

## 4. Materials and Methods

### 4.1. Collection of Phenotypic and Genotypic Data

The phenotypic and genotypic data used in this study were obtained from our previously published research [12]. In that study, mirror carp samples were collected from a commercial breeding population at the Hulan Breeding Station of the Heilongjiang River Fisheries Research Institute, Chinese Academy of Fishery Sciences. Following hatching, approximately 3000 larvae were reared in a pond, and approximately 1000 juveniles were randomly selected at around 60 days post hatching and individually tagged using passive integrated transponder tags (Biomark, Boise, ID, USA). The fish were subsequently reared under controlled conditions until two years of age, and approximately one third of the tagged individuals were randomly selected for phenotypic evaluation. Before measurement, the fish were anesthetized with 0.5 mg/L 2 phenoxyethanol (Sigma-Aldrich, St. Louis, MO, USA). Standard body length (SL) was measured using Vernier calipers (Mitutoyo, Kawasaki, Japan) with an accuracy of 0.1 cm and fillet fat content (FC) was measured using a Fish Fatmeter (Distell, Old Levenseat, Scotland, UK; Model FFM-692). Valid data were obtained from 325 individuals, and after excluding individuals with missing data, SL and FC data from 312 individuals were included in the analysis. Genomic DNA was extracted using a standard phenol chloroform method (Sigma-Aldrich, St. Louis, MO, USA), and DNA quality was assessed using a NanoDrop 2000C (Thermo Scientific, Waltham, MA, USA) before library preparation. Paired end sequencing was performed on the BGI T7 platform (BGI Group, Shenzhen, China) using the PE150 sequencing mode with an insert size of 350 bp and an average sequencing depth of 10×. Each sample generated approximately 17 Gb of raw sequencing data, with a total data volume of more than 5.5 Tb across the analyzed individuals. The data volume per sample was at least 85% of the expected yield, with a mean Q30 value of ≥88% and an effective conversion rate of ≥95%. Phenotypic distributions were visualized using the ggplot2 package (version 4.0.1) in R (version 4.4.2).

### 4.2. SNP Calling

The common carp reference genome (NCBI RefSeq: GCF_018340385.1, assembly version ASM1834038v1) was used as the reference for sequence alignment. Paired-end sequencing reads were aligned to the reference genome using the MEM algorithm implemented in BWA (version 0.7.17) [39]. The resulting alignments were sorted and PCR duplicates were marked using SAMtools (version 1.10) [40,41]. Subsequently, SNP calling was conducted using BCFtools (version 1.10.2). The mpileup command was used with a minimum mapping quality of −-q 20 and a minimum base quality of -Q 20, followed by genotype inference using the multiallelic calling model (-m) implemented in the call command to generate the raw VCF file [41,42]. High-confidence SNPs were retained using Bcftools filter, with thresholds set at a quality score ≥ 20, total sequencing depth ≥ 10, and mapping quality >20. PLINK (version 1.90b7.2) with the biallelic-only option was further used to retain only biallelic SNPs [43], and loci with a missing genotype rate > 0.2 and a minor allele frequency < 0.05 were removed, resulting in 24,752,633 SNP markers. To meet the marker number requirement of epistasis analysis (typically in the millions), these SNPs were first extracted using bcftools and sorted by chromosome and physical position. SNPs were then sequentially retained such that the physical distance between adjacent retained SNPs was at least 500 bp, resulting in 2,207,321 SNPs for subsequent analyses.

### 4.3. Detection of Epistatic Interactions

To investigate the epistatic interaction mechanisms affecting SL and FC traits in common carp, the Bridging Gene Sets with Epistasis (BridGE) tool (v2.0) was employed to perform pathway-level epistasis analysis [44]. The SNP set was first reduced by PLINK QC (--geno 0.02, --maf 0.05, --hwe 1e-6), LD pruning (--indep-pairwise 50 5 0.1), and D′-based redundancy filtering (50-SNP window; D′ > 0.5). Subsequently, SNPs within 50 kb upstream or downstream of annotated genes were assigned to genes based on the common carp reference genome (NCBI RefSeq GCF_018340385.1, ASM1834038v1). Pathways containing 10–300 genes were retained. Pairwise interaction scores were computed with CASSI v2.51 under a linear regression model [45]. Pairs with an interaction *p* < 0.1 were retained to build a sparse network. Ten phenotype-permuted datasets were generated in PLINK and processed using the same CASSI procedure. BridGE compared the real and permuted networks using Wilcoxon rank-sum tests to evaluate within-pathway model (WPM) and between-pathway model (BPM) enrichment, with 10,000 SNP permutations used to obtain empirical *p*-values. Network summaries were generated using a density cutoff of 0.1. The default BridGE reporting threshold of minFDR = 0.25 was not used for final candidate selection. No favourable candidate interaction signal satisfying the default minFDR criterion was identified in the WPM. Trait-specific candidate screening cut-offs were therefore defined at the minFDR values where interaction signals appeared in both WPM and BPM (0.55 for SL and 0.70 for FC). Based on these screening cut-offs, candidate epistatic interaction pathways, driver SNPs, and associated statistical information were retained for downstream analyses. For each retained interaction pair, the estimated coefficient of the interaction term was defined as the genetic interaction (GI) score.

### 4.4. Heritability Estimation

Genome-wide narrow-sense heritability ($h^{2}$) of SL and FC was estimated using the GCTA software (version 1.94) under the fastGWA-REML framework based on restricted maximum likelihood (GREML) [46]. A single-trait linear mixed model (LMM) was constructed to partition variance components as follows:

y =Xβ+ Zg + e

where y is the vector of phenotypic observations; β is a fixed-effect vector containing only the intercept; X and Z are incidence matrices for fixed and random effects, respectively; g is the random additive genomic effect vector assumed to follow $g\;\sim \;N\left(0,\sigma_{g}^{2}K\right)$, where K is the genomic relationship matrix (GRM) constructed using the standard VanRaden method 2 [47]; and e is the residual error vector assumed to follow $e\;\sim \;N\left(0,\sigma_{e}^{2}I\right)$. Based on the estimated additive genetic variance ($\sigma_{g}^{2}$) and residual variance ($\sigma_{e}^{2}$), narrow-sense heritability was calculated as:

$$h^{2}\;=\;\frac{\sigma_{g}^{2}}{\sigma_{g}^{2}\;+\;\sigma_{e}^{2}}$$

The significance of heritability estimates was evaluated using *p*-values derived from likelihood ratio tests.

### 4.5. Integration of Genomic Selection Models

In this study, six baseline models were employed to perform GS analyses for SL and FC traits in common carp. These models included four Bayesian approaches: BayesA, BayesB, BayesC, and Bayes LASSO (BL) [48,49,50], as well as two linear mixed models: Ridge Regression Best Linear Unbiased Prediction (RRBLUP) and Genomic Best Linear Unbiased Prediction (GBLUP) [51,52]. To objectively evaluate the marginal contribution of epistatic effects, the additive model and the additive-epistatic model utilized the identical additive design matrix ($X_{\mathrm{add}}$). This matrix was constructed from the filtered 12,902 SNPs and subjected to mean imputation and centering (scale = FALSE). Model analyses were performed using the BGLR package (v1.1.4) in R [53], with the MCMC parameters configured to nIter = 12,000, burnIn = 4000, and thin = 5. To investigate the impact of epistatic interactions on genomic predictive ability, driver SNP pairs identified from the BridGE pipeline were selected based on their favourable effects. A two-stage explicit matrix construction strategy was applied to model these interactions. Epistatic pseudo-markers were constructed for each candidate SNP pair by taking the Hadamard product ($X_{\mathrm{epi}}\;=\;X_{\mathrm{SNP}1}\;⊙\;X_{\mathrm{SNP}2}$) of standardized allele dosage vectors (0, 1, 2). This setup captures only pairwise additive × additive effects and excludes dominance and higher-order interactions. The resulting vectors were compiled into a standalone epistatic matrix ($M_{\mathrm{epi}}$) and fitted in BGLR as an independent random effect, parallel to the main additive genomic term. Model evaluation relied on 50 repetitions of five-fold cross-validation. Within each repeat, samples were randomly partitioned into five approximately equal folds. A fixed seed (set.seed(123)) ensured that all models shared identical validation splits. In each cycle, four folds were used for training and one for testing, yielding 250 predictions per model. Predictive ability was defined as the mean Pearson correlation between predicted and observed phenotypes across the 250 predictions. For the equal-density comparison, ten random subsets of 12,902 SNPs were drawn from the initial 2,207,321 SNPs. Each subset was analyzed with the additive GBLUP model using the same cross-validation partitions and BGLR settings as the epistasis-informed panel. The mean ± SD predictive ability across the ten subsets was reported. Results were visualized in R using ggplot2 (v4.0.1).

### 4.6. AlphaFold2 Prediction and Structural Visualization

To obtain complementary structural information for the interaction relationships identified by the BridGE algorithm, interaction pairs associated with SL and FC were subjected to favourable effect screening and gene-level deduplication. The non-redundant favourable interaction pairs obtained after this filtering process were defined as driver interaction pairs in this study, and AlphaFold2 (AF2) was used to predict their protein complex structures [21]. The structural prediction of the AF2 protein complex was performed using a locally deployed Protein Server platform with the underlying model version proteinx_base_20250630_v1.0.0 [54]. This model implements a deep-learning architecture equivalent to AlphaFold-Multimer. Multiple sequence alignment and template search were enabled during prediction. Five candidate models were generated for each target, with the recycle number set to 10, the diffusion step number set to 200, and the random seed set to 70,000. Among the five candidate models, the model with the highest ranking_score was selected as the representative structure, where the ranking_score was calculated from a weighted combination of ipTM and pTM. Subsequently, for each trait, the top 1000 interaction pairs with the highest GI scores were selected from the driver interaction pairs, and the full-length amino acid sequences of the encoded proteins were extracted as the experimental dataset. Meanwhile, 1000 length-matched protein pairs were randomly sampled from the common carp reference genome protein FASTA file as a size-matched baseline. Length matching helps control for a major technical driver of ipTM, but this baseline was not assumed to represent true non-interacting pairs. AF2 was then used for PPI prediction and three-dimensional structural modeling, and the predicted interactions were evaluated using the interface predicted TM-score (ipTM) [55,56]. The predicted Local Distance Difference Test (pLDDT) values were extracted from the plddt field in the corresponding JSON output files and the associated visualization output. A protein pair was considered successfully modeled when both protein chains had complete all-atom coordinates, no severe structural clashes were present, and confidence assessment metrics were successfully generated. Based on these results, the three interaction pairs with the highest ipTM scores for each trait were visualized using ChimeraX (v1.7.1) to illustrate the structural characteristics of their protein interaction interfaces [57]. Buried surface area (BSA) was also calculated in UCSF ChimeraX v1.7.1, based on the solvent-accessible surface area (SASA) of individual chains and the assembled complex. Calculations used a standard probe radius of 1.4 Å, following the formula: BSA = (SASA_A + SASA_B - SASA_AB)∕2.

### 4.7. Gene Functional Enrichment and Protein Interaction Network Analysis

Functional and Gene Ontology (GO) enrichment analyses were performed for genes involved in the driver interaction pairs using the agriGO platform [58]. For the target gene set, pathway significance was evaluated using Fisher’s exact test, and *p*-values were adjusted for multiple testing using the Benjamini-Yekutieli method to control the false discovery rate (FDR) [59]. Enrichment results were visualized using the ggplot2 package (v4.0.1) in R. To further explore the physical interactions among core driver factors, a PPI network was constructed based on the subset of driver interaction pairs with ipTM ≥ 0.60 from the AF2 predictions. Network visualization was performed using the tidygraph (v1.3.1) and ggraph (v2.2.1) packages in R. In this network, genes were represented as nodes, with node size proportional to connectivity to reflect hub importance. Edges represented gene–gene interactions, with edge width and color encoding GI values and subgroup classification, respectively. A circular layout was used, and an integrated circular heatmap combining average GI and average ipTM values for each gene was generated. Network visualization was implemented using the R packages tidygraph (v 1.3.1), ggraph (v 2.2.1), and ggnewscale (v 0.5.2).

### 4.8. Two-Locus Genotype Combination Analysis

To evaluate phenotypic differences associated with candidate interaction pairs, we selected the three candidate pairs with the highest GI scores for each trait. Genotypes at the two interacting loci were extracted and combined into two-locus genotype combinations. Given that the linkage disequilibrium between the loci was low (r^2^ < 0.1), these combinations were analyzed directly rather than as phased haplotypes. Phenotypic differences among combinations were assessed using one-way ANOVA followed by Tukey’s HSD test (*p* < 0.05). Pairwise comparisons were restricted to combinations with *n* ≥ 5. Kruskal–Wallis tests were also performed as sensitivity checks. In these tests, low-frequency combinations were pooled into a single category. Sample sizes for all two-locus genotype combinations are given in Table S4. Data manipulation and plotting were performed using dplyr (v1.1.4) and ggplot2 (v4.0.1) in R.

## 5. Conclusions

In this study, we used standard body length (SL) and fillet fat content (FC) in common carp as target traits to investigate the potential contribution of epistatic effects to the genetic architecture of complex traits. Our results identified candidate epistatic interactions associated with both traits. The SL trait shows a moderate additive genetic contribution, whereas the additive genetic variance of FC is extremely limited. Under the present analytical framework, incorporating selected epistatic effects into GS models was associated with higher predictive ability for both traits. AlphaFold2 protein structure prediction provided computational structural support for a subset of candidate driver interaction pairs. Integrated analyses of interaction networks, functional enrichment, and two-locus genotype–phenotype associations provided additional information for candidate prioritization. These analyses also suggested several candidate mechanisms, including protein complex cooperation, pathway response coordination, and genotype combination effects. Overall, these findings support the potential contribution of epistatic information to genomic prediction in common carp and provide a basis for further evaluation in independent populations.

## Figures and Tables

**Table 1:** Estimation of additive genetic parameters for Standard body length (SL) and Fillet fat content (FC) in 312 common carp. The table presents narrow-sense heritability (h^2^), likelihood ratio test significance (*p*-value), additive genetic variance (Vg = $\sigma_{g}^{2}$), and environmental variance (Ve = $\sigma_{e}^{2}$) for each trait. Values marked with * indicate statistical significance at the 0.05 level.

Trait	Heritability	*p*-Value	Vg	Ve
SL	0.34	0.01 *	2.79 ± 1.10	5.49 ± 0.83
FC	0.00	1.00	3.48 × 10^-18^ ± 0.01	0.06 ± 0.01

## Data Availability

The raw sequencing data generated in this study are now deposited in the BioProject database of the National Genomics Data Center (NGDC) under accession number PRJCA037933. Furthermore, the intermediate datasets have been made available via Zenodo at https://doi.org/10.5281/zenodo.22182862.

## Supplementary Materials

The following supporting information can be downloaded at: https://www.mdpi.com/article/10.3390/ijms27188315/s1.
