# Supplementary material for: Integrating Epistatic Interactions into Genomic Prediction of Growth and Fillet Fat Content in Common Carp
Source: Int J Mol Sci. 2026 Sep 18;27(18):8315. doi: 10.3390/ijms27188315 (PMC13607738; doi:10.3390/ijms27188315)
Supplement: Supplementary file 1 [file ijms-27-08315-s001.zip › Supplementary_Figure S1.pdf]

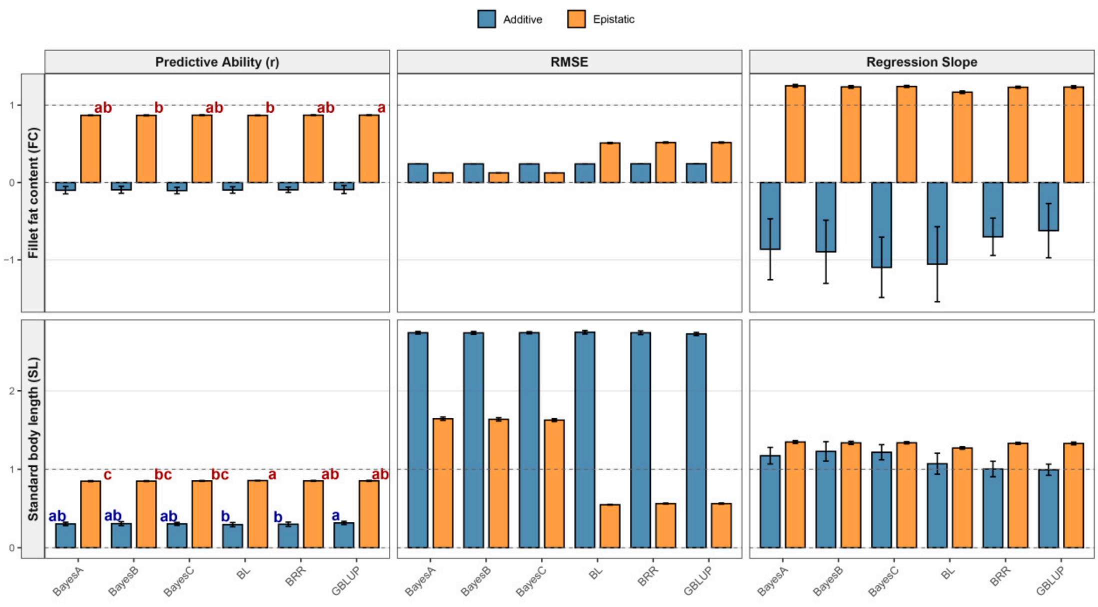

Figure S1. Comprehensive evaluation of genomic selection (GS) prediction performance across different models.

Bar plots show the prediction performance metrics (Predictive Ability (r), Root Mean Square Error (RMSE), and Regression Slope) for six genomic selection models (BayesA, BayesB, BayesC, Bayes LASSO, RRBLUP, and GBLUP) under two effect frameworks, evaluated for standard body length (SL) and fillet fat content (FC). “Additive” (blue) represents baseline models including only additive effects, while “Epistatic” (orange) represents models incorporating epistatic SNP interactions. Data are presented as mean ± SD across 50 cross-validation replicates (N = 312). The error bars reflect the variability across repetitions. In the Predictive Ability panels, different lowercase letters (blue for Additive, red for Epistatic) indicate statistically significant differences among models within each framework, as determined by one-way ANOVA followed by Tukey’s HSD test ($P < 0.05$). Note that all models exhibited a prediction bias close to zero (ranging from -0.007 to 0.007); therefore, the bias metric is not shown in this figure.
